# Large scale multifactorial likelihood quantitative analysis of *BRCA1* and *BRCA2* variants: An ENIGMA resource to support clinical variant classification

**DOI:** 10.1002/humu.23818

**Published:** 2019-09-13

**Authors:** Michael T. Parsons, Emma Tudini, Hongyan Li, Eric Hahnen, Barbara Wappenschmidt, Lidia Feliubadaló, Cora M. Aalfs, Simona Agata, Kristiina Aittomäki, Elisa Alducci, María Concepción Alonso‐Cerezo, Norbert Arnold, Bernd Auber, Rachel Austin, Jacopo Azzollini, Judith Balmaña, Elena Barbieri, Claus R. Bartram, Ana Blanco, Britta Blümcke, Sandra Bonache, Bernardo Bonanni, Åke Borg, Beatrice Bortesi, Joan Brunet, Carla Bruzzone, Karolin Bucksch, Giulia Cagnoli, Trinidad Caldés, Almuth Caliebe, Maria A. Caligo, Mariarosaria Calvello, Gabriele L. Capone, Sandrine M. Caputo, Ileana Carnevali, Estela Carrasco, Virginie Caux‐Moncoutier, Pietro Cavalli, Giulia Cini, Edward M. Clarke, Paola Concolino, Elisa J. Cops, Laura Cortesi, Fergus J. Couch, Esther Darder, Miguel de la Hoya, Michael Dean, Irmgard Debatin, Jesús Del Valle, Capucine Delnatte, Nicolas Derive, Orland Diez, Nina Ditsch, Susan M. Domchek, Véronique Dutrannoy, Diana M. Eccles, Hans Ehrencrona, Ute Enders, D. Gareth Evans, Chantal Farra, Ulrike Faust, Ute Felbor, Irene Feroce, Miriam Fine, William D. Foulkes, Henrique C.R. Galvao, Gaetana Gambino, Andrea Gehrig, Francesca Gensini, Anne‐Marie Gerdes, Aldo Germani, Jutta Giesecke, Viviana Gismondi, Carolina Gómez, Encarna B. Gómez Garcia, Sara González, Elia Grau, Sabine Grill, Eva Gross, Aliana Guerrieri‐Gonzaga, Marine Guillaud‐Bataille, Sara Gutiérrez‐Enríquez, Thomas Haaf, Karl Hackmann, Thomas V.O. Hansen, Marion Harris, Jan Hauke, Tilman Heinrich, Heide Hellebrand, Karen N. Herold, Ellen Honisch, Judit Horvath, Claude Houdayer, Verena Hübbel, Silvia Iglesias, Angel Izquierdo, Paul A. James, Linda A.M. Janssen, Udo Jeschke, Silke Kaulfuß, Katharina Keupp, Marion Kiechle, Alexandra Kölbl, Sophie Krieger, Torben A. Kruse, Anders Kvist, Fiona Lalloo, Mirjam Larsen, Vanessa L. Lattimore, Charlotte Lautrup, Susanne Ledig, Elena Leinert, Alexandra L. Lewis, Joanna Lim, Markus Loeffler, Adrià López‐Fernández, Emanuela Lucci‐Cordisco, Nicolai Maass, Siranoush Manoukian, Monica Marabelli, Laura Matricardi, Alfons Meindl, Rodrigo D. Michelli, Setareh Moghadasi, Alejandro Moles‐Fernández, Marco Montagna, Gemma Montalban, Alvaro N. Monteiro, Eva Montes, Luigi Mori, Lidia Moserle, Clemens R. Müller, Christoph Mundhenke, Nadia Naldi, Katherine L. Nathanson, Matilde Navarro, Heli Nevanlinna, Cassandra B. Nichols, Dieter Niederacher, Henriette R. Nielsen, Kai‐ren Ong, Nicholas Pachter, Edenir I. Palmero, Laura Papi, Inge Sokilde Pedersen, Bernard Peissel, Pedro Perez‐Segura, Katharina Pfeifer, Marta Pineda, Esther Pohl‐Rescigno, Nicola K. Poplawski, Berardino Porfirio, Anne S. Quante, Juliane Ramser, Rui M. Reis, Françoise Revillion, Kerstin Rhiem, Barbara Riboli, Julia Ritter, Daniela Rivera, Paula Rofes, Andreas Rump, Monica Salinas, Ana María Sánchez de Abajo, Gunnar Schmidt, Ulrike Schoenwiese, Jochen Seggewiß, Ares Solanes, Doris Steinemann, Mathias Stiller, Dominique Stoppa‐Lyonnet, Kelly J. Sullivan, Rachel Susman, Christian Sutter, Sean V. Tavtigian, Soo H. Teo, Alex Teulé, Mads Thomassen, Maria Grazia Tibiletti, Marc Tischkowitz, Silvia Tognazzo, Amanda E. Toland, Eva Tornero, Therese Törngren, Sara Torres‐Esquius, Angela Toss, Alison H. Trainer, Katherine M. Tucker, Christi J. van Asperen, Marion T. van Mackelenbergh, Liliana Varesco, Gardenia Vargas‐Parra, Raymonda Varon, Ana Vega, Ángela Velasco, Anne‐Sophie Vesper, Alessandra Viel, Maaike P. G. Vreeswijk, Sebastian A. Wagner, Anke Waha, Logan C. Walker, Rhiannon J. Walters, Shan Wang‐Gohrke, Bernhard H. F. Weber, Wilko Weichert, Kerstin Wieland, Lisa Wiesmüller, Isabell Witzel, Achim Wöckel, Emma R. Woodward, Silke Zachariae, Valentina Zampiga, Christine Zeder‐Göß, KConFab Investigators, Conxi Lázaro, Arcangela De Nicolo, Paolo Radice, Christoph Engel, Rita K. Schmutzler, David E. Goldgar, Amanda B. Spurdle

**Affiliations:** ^1^ Department of Genetics and Computational Biology QIMR Berghofer Medical Research Institute Brisbane Queensland Australia; ^2^ Cancer Control and Population Science, Huntsman Cancer Institute University of Utah Salt Lake City Utah; ^3^ Center for Familial Breast and Ovarian Cancer, Faculty of Medicine and University Hospital Cologne University of Cologne Cologne Germany; ^4^ Center for Integrated Oncology (CIO), Faculty of Medicine and University Hospital Cologne University of Cologne Cologne Germany; ^5^ Hereditary Cancer Program, ONCOBELL‐IDIBELL‐IDIBGI‐IGTP, Catalan Institute of Oncology CIBERONC Barcelona Spain; ^6^ Department of Clinical Genetics Amsterdam UMC Amsterdam The Netherlands; ^7^ Immunology and Molecular Oncology Unit, Veneto Institute of Oncology IOV IRCCS Padua Italy; ^8^ Department of Clinical Genetics, Helsinki University Hospital University of Helsinki Helsinki Finland; ^9^ Clinical Genetics, Hospital Universitario de la Princesa Instituto de Investigación Sanitaria Madrid Spain; ^10^ Department of Gynaecology and Obstetrics, University Hospital of Schleswig‐Holstein, Campus Kiel Christian‐Albrechts University Kiel Kiel Germany; ^11^ Institute of Clinical Molecular Biology, University Hospital of Schleswig‐Holstein, Campus Kiel Christian‐Albrechts University Kiel Kiel Germany; ^12^ Institute of Human Genetics Hannover Medical School Hannover Germany; ^13^ Genetic Health Queensland Royal Brisbane and Women's Hospital Brisbane Australia; ^14^ Unit of Medical Genetics, Department of Medical Oncology and Hematology Fondazione IRCCS Istituto Nazionale dei Tumori di Milano Milan Italy; ^15^ High Risk and Cancer Prevention Group Vall d'Hebron Institute of Oncology Barcelona Spain; ^16^ Department of Medical Oncology University Hospital of Vall d'Hebron Barcelona Spain; ^17^ Department of Oncology and Haematology University of Modena and Reggio Emilia Modena Italy; ^18^ Institute of Human Genetics University Hospital Heidelberg Heidelberg Germany; ^19^ Fundación Pública galega Medicina Xenómica‐SERGAS Grupo de Medicina Xenómica‐USC, CIBERER, IDIS Santiago de Compostela Spain; ^20^ Oncogenetics Group Vall d'Hebron Institute of Oncology (VHIO) Barcelona Spain; ^21^ Division of Cancer Prevention and Genetics, IEO European Institute of Oncology IRCCS Milan Italy; ^22^ Division of Oncology and Pathology, Department of Clinical Sciences Lund Lund University Lund Sweden; ^23^ Division of Oncology University Hospital of Parma Parma Italy; ^24^ Unit of Hereditary Cancer IRCCS Ospedale Policlinico San Martino Genoa Italy; ^25^ Institute for Medical Informatics, Statistics and Epidemiology University of Leipzig Leipzig Germany; ^26^ Molecular Oncology Laboratory, CIBERONC, Hospital Clinico San Carlos IdISSC (Instituto de Investigación Sanitaria del Hospital Clínico San Carlos) Madrid Spain; ^27^ Institute of Human Genetics, University Hospital of Schleswig‐Holstein, Campus Kiel Christian‐Albrechts University Kiel Kiel Germany; ^28^ SOD Genetica Molecolare University Hospital Pisa Italy; ^29^ Department of Experimental and Clinical Biomedical Sciences 'Mario Serio', Medical Genetics Unit University of Florence Florence Italy; ^30^ Service de Génétique Institut Curie Paris France; ^31^ Paris Sciences Lettres Research University Paris France; ^32^ UO Anatomia Patologica Ospedale di Circolo ASST Settelaghi Varese Italy; ^33^ Servizio di Genetica ASST di Cremona Cremona Italy; ^34^ Division of Functional Onco‐genomics and Genetics, Centro di Riferimento Oncologico di Aviano (CRO) IRCCS Aviano Italy; ^35^ Fondazione Policlinico Universitario A.Gemelli IRCCS Rome Italy; ^36^ Parkville Familial Cancer Centre Peter MacCallum Cancer Center Melbourne Victoria Australia; ^37^ Department of Laboratory Medicine and Pathology Mayo Clinic Rochester Minnesota; ^38^ Laboratory of Translational Genomics, DCEG National Cancer Institute Gaithersburg Maryland; ^39^ Institute of Human Genetics University Hospital Ulm Ulm Germany; ^40^ Laboratoire de Genetique Moleculaire CHU Nantes Nantes France; ^41^ Clinical and Molecular Genetics Area University Hospital Vall d'Hebron Barcelona Spain; ^42^ Department of Gynecology and Obstetrics University of Munich Munich Germany; ^43^ Basser Center for BRCA, Abramson Cancer Center University of Pennsylvania Philadelphia Pennsylvania; ^44^ Institute of Medical and Human Genetics Charité –Universitätsmedizin Berlin Berlin Germany; ^45^ Faculty of Medicine University of Southampton Southampton UK; ^46^ Department of Clinical Genetics and Pathology, Laboratory Medicine Office for Medical Services ‐ Region Skåne Lund Sweden; ^47^ Division of Clinical Genetics, Department of Laboratory Medicine Lund University Lund Sweden; ^48^ Genomic Medicine, Division of Evolution and Genomic Sciences, The University of Manchester, Manchester Academic Health Science Centre, Manchester Universities Foundation Trust St. Mary's Hospital Manchester UK; ^49^ Genomic Medicine, North West Genomics hub, Manchester Academic Health Science Centre, Manchester Universities Foundation Trust St. Mary's Hospital Manchester UK; ^50^ Medical Genetics American University of Beirut Medical Center Beirut Lebanon; ^51^ Institute of Medical Genetics and Applied Genomics University of Tübingen Tübingen Germany; ^52^ Institute of Human Genetics University Medicine Greifswald Greifswald Germany; ^53^ Adult Genetics Unit Royal Adelaide Hospital Adelaide Australia; ^54^ Program in Cancer Genetics, Departments of Human Genetics and Oncology McGill University Montréal QC Canada; ^55^ Oncogenetics Department Barretos Cancer Hospital São Paulo Brazil; ^56^ Department of Human Genetics University of Würzburg Würzburg Germany; ^57^ Department of Clinical Genetics, Rigshospitalet Copenhagen University Hospital Copenhagen Denmark; ^58^ Department of Clinical and Molecular Medicine, Sant'Andrea University Hospital Sapienza University Rome Italy; ^59^ Department of Clinical Genetics Maastricht University Medical Center Maastricht The Netherlands; ^60^ Division of Gynaecology and Obstetrics, Klinikum rechts der Isar der Technischen Universität München Munich Germany; ^61^ Departement de Biopathologie, Gustave Roussy Universite Paris‐Saclay Villejuif France; ^62^ Institute for Clinical Genetics, Faculty of Medicine Carl Gustav Carus TU Dresden Dresden Germany; ^63^ Familial Cancer Centre Monash Health Clayton Australia; ^64^ The City of Hope Duarte California; ^65^ Department of Gynecology and Obstetrics, University Hospital Düsseldorf Heinrich‐Heine University Düsseldorf Düsseldorf Germany; ^66^ Institute of Human Genetics University of Münster Münster Germany; ^67^ Department of Genetics, F76000 and Normandy University, UNIROUEN, Inserm U1245, Normandy Centre for Genomic and Personalized Medicine Rouen University Hospital Rouen France; ^68^ Sir Peter MacCallum Department of Oncology The University of Melbourne Melbourne Victoria Australia; ^69^ Department of Clinical Genetics Leiden University Medical Center Leiden The Netherlands; ^70^ Institute of Human Genetics University Medical Center Göttingen Göttingen Germany; ^71^ Laboratoire de Biologie Clinique et Oncologique Centre Francois Baclesse Caen France; ^72^ Genomics and Personalized Medecine in Cancer and Neurological Disorders Normandy Centre for Genomic and Personalized Medicine Rouen France; ^73^ Normandie Université UNICAEN Caen France; ^74^ Department of Clinical Genetics Odense University Hospital Odense C Denmark; ^75^ Department of Pathology and Biomedical Science University of Otago Christchurch New Zealand; ^76^ Department of Clinical Genetics Aalborg University Hospital Aalborg Denmark; ^77^ Clinical Cancer Research Center Aalborg University Hospital Aalborg Denmark; ^78^ Department of Gynaecology and Obstetrics University Hospital Ulm Ulm Germany; ^79^ Peter MacCallum Cancer Centre Melbourne Victoria Australia; ^80^ Breast Cancer Research Programme Cancer Research Malaysia Subang Jaya Selangor Malaysia; ^81^ UOC Genetica Medica, Fondazione Policlinico Universitario A.Gemelli IRCCS and Istituto di Medicina Genomica Università Cattolica del Sacro Cuore Rome Italy; ^82^ Department of Cancer Epidemiology Moffitt Cancer Center Tampa Florida; ^83^ Department of Clinical and Experimental Science, University of Brescia c/o 2nd Internal Medicine Hospital of Brescia Brescia Italy; ^84^ Department of Obstetrics and Gynecology, Helsinki University Hospital University of Helsinki Helsinki Finland; ^85^ Genetic Services of Western Australia King Edward Memorial Hospital Perth Australia; ^86^ West Midlands Regional Genetics Service Birmingham Women's Hospital Healthcare NHS Trust Birmingham UK; ^87^ Faculty of Health and Medical Sciences University of Western Australia Perth Australia; ^88^ Molecular Oncology Research Center Barretos Cancer Hospital São Paulo Brazil; ^89^ Barretos School of Health Sciences Dr. Paulo Prata ‐ FACISB São Paulo Brazil; ^90^ Molecular Diagnostics Aalborg University Hospital Aalborg Denmark; ^91^ Department of Clinical Medicine Aalborg University Aalborg Denmark; ^92^ School of Paediatrics and Reproductive Health University of Adelaide Adelaide Australia; ^93^ Health Sciences School University of Minho Braga Portugal; ^94^ ICVS/3B's‐PT Government Associate Laboratory Braga Portugal; ^95^ Laboratoire d'Oncogenetique Moleculaire Humaine Centre Oscar Lambret Lille France; ^96^ Servicio de Análisis Clínicos y Bioquímica Clínica, Complejo Hospitalario Universitario Insular Materno‐Infantil de Gran Canaria Las Palmas de Gran Canaría Spain; ^97^ Institute of Human Genetics University Hospital Leipzig Leipzig Germany; ^98^ Department of Tumour Biology INSERM U830 Paris France; ^99^ Université Paris Descartes Paris France; ^100^ Genetic Health Service NZ‐ Northern Hub Auckland District Health Board Auckland New Zealand; ^101^ Department of Oncological Services University of Utah School of Medicine Salt Lake City Utah; ^102^ Huntsman Cancer Institute University of Utah Salt Lake City Utah; ^103^ Department of Surgery, Faculty of Medicine University Malaya Kuala Lumpur Malaysia; ^104^ Department of Medical Genetics University of Cambridge Cambridge UK; ^105^ Department of Cancer Biology and Genetics The Ohio State University Columbus Ohio; ^106^ Department of medicine University of Melbourne Melbourne Victoria Australia; ^107^ Prince of Wales Clinical School University of NSW Sydney New South Wales Australia; ^108^ Hereditary Cancer Clinic, Department of Medical Oncology Prince of Wales Hospital Randwick New South Wales Australia; ^109^ Department of Human Genetics Leiden University Medical Center Leiden The Netherlands; ^110^ Department of Medicine Hematology/Oncology, Goethe‐University Frankfurt Frankfurt Germany; ^111^ Institute of Human Genetics University Regensburg Regensberg Germany; ^112^ Institute of Pathology Technische Universität München Munich Germany; ^113^ Department of Gynecology University Medical Center Hamburg Hamburg Germany; ^114^ Department of Gynecology and Obstetrics University Hospital Würzburg Würzburg Germany; ^115^ Biosciences Laboratory Istituto Scientifico Romagnolo per lo Studio e la Cura dei Tumori (IRST) IRCCS Meldola Italy; ^116^ Research Department Peter MacCallum Cancer Center Melbourne Victoria Australia; ^117^ Cancer Genomics Program Veneto Institute of Oncology IOV‐IRCCS Padua Italy; ^118^ Unit of Molecular Bases of Genetic Risk and Genetic Testing, Department of Research Fondazione IRCCS Istituto Nazionale dei Tumori (INT) Milan Italy; ^119^ Department of Dermatology, Huntsman Cancer Institute University of Utah School of Medicine Salt Lake City Utah

**Keywords:** BRCA1, BRCA2, classification, clinical, multifactorial, quantitative, uncertain significance, variant

## Abstract

The multifactorial likelihood analysis method has demonstrated utility for quantitative assessment of variant pathogenicity for multiple cancer syndrome genes. Independent data types currently incorporated in the model for assessing *BRCA1* and *BRCA2* variants include clinically calibrated prior probability of pathogenicity based on variant location and bioinformatic prediction of variant effect, co‐segregation, family cancer history profile, co‐occurrence with a pathogenic variant in the same gene, breast tumor pathology, and case‐control information. Research and clinical data for multifactorial likelihood analysis were collated for 1,395 *BRCA1/2* predominantly intronic and missense variants, enabling classification based on posterior probability of pathogenicity for 734 variants: 447 variants were classified as (likely) benign, and 94 as (likely) pathogenic; and 248 classifications were new or considerably altered relative to ClinVar submissions. Classifications were compared with information not yet included in the likelihood model, and evidence strengths aligned to those recommended for ACMG/AMP classification codes. Altered mRNA splicing or function relative to known nonpathogenic variant controls were moderately to strongly predictive of variant pathogenicity. Variant absence in population datasets provided supporting evidence for variant pathogenicity. These findings have direct relevance for *BRCA1* and *BRCA2* variant evaluation, and justify the need for gene‐specific calibration of evidence types used for variant classification.

## 1 INTRODUCTION

*BRCA1* and *BRCA2* variants resulting in abrogated function of the encoded proteins confer a high risk of breast and ovarian cancer (Antoniou et al., 2003; Kuchenbaecker et al., 2017), and have been reported to increase risk of several other cancer types (Breast Cancer Linkage C, 1999; Ford, Easton, Bishop, Narod, & Goldgar, 1994; Moran et al., 2012; Thompson, Easton, & Breast Cancer Linkage C, 2002). The cancer types commonly considered important for risk assessment are breast (female and male), ovarian, prostate, and pancreatic cancer, all of which are included in the BOADICEA model predicting risk of cancer for *BRCA1* and *BRCA2* pathogenic variant carriers (Antoniou et al., 2008). Identification of a pathogenic *BRCA1* or *BRCA2* variant is important clinical information that directs medical management of an individual, including strategies aimed at prevention (risk‐reducing surgery or medication), early detection (presymptomatic screening), and more recently personalized treatment with PARP‐inhibitors (Pilie, Tang, Mills, & Yap, 2019). Further, cascade testing of close relatives of a pathogenic variant carrier is an efficient and cost‐effective way to reduce the burden of cancer in individuals at high risk of developing cancer (Tuffaha et al., 2018). However, *BRCA1* and *BRCA2* variants of uncertain clinical significance (VUS) identified by diagnostic testing continue to pose a challenge for management of patients and their relatives.

ENIGMA (Evidence‐based Network for the Interpretation of Germline Mutant Alleles) is an international research consortium focused on developing and applying methods to determine the clinical significance in breast‐ovarian cancer predisposition genes (Spurdle et al., 2012). ENIGMA has developed variant classification criteria that utilize both quantitative (statistical) and qualitative (rules‐based) methods to assess the clinical significance of variants in *BRCA1* and *BRCA2* (http://enigmaconsortium.org/). Quantitative classifications of variants by ENIGMA are derived from the multifactorial likelihood model (Goldgar et al., 2004; Goldgar et al., 2008) that combines multiple lines of clinical data in a Bayesian framework, with the assumption that each feature is an independent predictor of variant pathogenicity. The *BRCA1/2* model components include likelihood ratios (LRs) for pathogenicity estimated from clinical data, such as co‐segregation with disease, co‐occurrence with a pathogenic variant in the same gene, reported family history, breast tumor pathology, and more recently, case‐control data (de la Hoya et al., 2016; Easton et al., 2007; Goldgar et al., 2008; Spurdle et al., 2014; Thompson, Easton, & Goldgar, 2003). This information is combined with a prior probability of pathogenicity based on bioinformatic predictions of variant effect on protein sequence or messenger RNA (mRNA) splicing (Tavtigian, Byrnes, Goldgar, & Thomas, 2008; Vallee et al., 2016), probabilities that have been calibrated against clinical information, to produce a quantitative classification applicable across many variant types. It should be noted that the reference sets used to derive estimates of these LRs and prior probabilities were selected such that the model is designed to assess if a variant demonstrates the clinical features observed for a classical “high risk” variant. To date there are 297 entries on the *BRCA1/2* database displaying variants classified by this method (http://hci‐exlovd.hci.utah.edu/home.php), and for which there has been publication of the breakdown of LRs for each component.

The American College of Medical Genetics and Genomics (ACMG) and Association for Molecular Pathology (AMP) developed a formal framework for using qualitative criteria for variant classification, in an attempt to standardize the application of such evidence (Richards et al., 2015). These guidelines are intended to be generic, and thus some evidence codes will not be relevant for variant curation for a specific gene. The ClinGen consortium (Rehm et al., 2015; https://www.clinicalgenome.org/) has engaged with expert groups to develop adaptations of the guidelines to specify which rule codes and strengths are appropriate for a specific gene‐disease relationship, and to provide guidance on the phenotypic features that are most predictive of variant pathogenicity (Rivera‐Munoz et al., 2018). To date, adaptations of the ACMG/AMP criteria have been completed for two hereditary cancer genes: *PTEN* (Mester et al., 2018) and *CDH1* (Lee et al., 2018), whereas other gene‐adaptations are in development. In addition, ClinGen has approved two additional expert panels arising from pre‐existing international research consortia, for the curation of variants in the mismatch repair genes (InSiGHT, International Society for Gastrointestinal Hereditary Tumours; https://www.insight‐group.org), and in *BRCA1* and *BRCA2* (ENIGMA; http://enigmaconsortium.org/). The classification criteria used by these groups pre‐dated the development of ACMG/AMP adaptations, and use quantitative methods (as noted above) and qualitative criteria not yet cross‐mapped to ACMG/AMP codes. The ClinGen Sequence Variant Interpretation Working Group recently demonstrated that the ACMG/AMP criteria were broadly compatible with Bayesian statistical reasoning, and estimated LR ranges appropriate for code strengths (Tavtigian et al., 2018).

Reference data is essential to calibrate the appropriate gene‐specific strength of different curation evidence types, and also to assess if evidence strength is the same for variants with different molecular effects. In relation to *BRCA1* and *BRCA2,* most variants established to be pathogenic are premature truncation variants (including nonsense or frameshift), and thus comprise the majority of variants in reference sets used to calibrate predictors of *BRCA1/2* variant pathogenicity. We have collated data from >40 clinical sites within ENIGMA and from other international clinical collaborators to conduct the largest application of the multifactorial likelihood model to missense and intronic variants in *BRCA1* and *BRCA2*, providing quantitative variant classifications, and also scores for the component data types so as to demonstrate their relative contributions to the final posterior probability. We have also demonstrated the value of this dataset as a resource for calibrating qualitative information for application to *BRCA1/2* variant classification, and deriving *BRCA1/2*‐appropriate rule strengths for several ACMG/AMP evidence codes.

## 2 METHODS

### 2.1 Variant selection for data collection

At the time of joining ENIGMA, members were asked to submit all variants in *BRCA1* and *BRCA2* that they considered to be of uncertain clinical significance, together with the number of families carrying each variant. We followed the rationale that high‐risk variants will not occur commonly in the general population, as indicated by frequency measured in outbred reference datasets representative of subpopulations. Variants were thus classified as Class 1 Not Pathogenic if they were identified to occur at minor allele frequency >0.01 in one or more of the following datasets: South Asian, Latino, African, East Asian, Non‐Finnish European subpopulations from the Exome Aggregation Consortium (*ExAC*) dataset (after excluding cancer‐related information from The Cancer Genome Atlas (TCGA; http://exac.broadinstitute.org); European, African, Admixed American, East Asian, or South Asian sample sets from the 1000 Genomes Project (http://www.1000genomes.org). This exercise provided a baseline variant list for subsequent ENIGMA studies. For the analysis presented in this study, a subset of variants were prioritized for collection of segregation and breast tumor pathology data, based on number of observations/families in the initial ENIGMA variant list and/or bioinformatic score indicative of pathogenicity. Information for co‐segregation analysis was provided in the form of a deidentified pedigree for families with known carrier status in more than one individual. Pedigree details included sex, cancer status, and age at cancer diagnosis, or age at interview if unaffected. Unaffected individuals known to have undergone prophylactic surgery (mastectomy or oophorectomy) were censored at age of earliest surgery. Breast tumor pathology information collected for known variant carriers included hormone receptor status (estrogen receptor [ER], progesterone receptor [PR], human epidermal growth factor receptor 2 [HER2]), and/or tumor grade. In addition, clinical queries to the Spurdle laboratory led to the collation of additional segregation and pathology information of potential value for multifactorial likelihood analysis of individual variants. Further, genotype data generated as part of the iCOGS project was available via collaboration with the Breast Cancer Association Consortium (BCAC) for a subset of variants, from up to 41,141 breast cancer cases and 38,694 controls of European ancestry, and 6,185 breast cancer cases and 6,614 controls of Asian ancestry (Michailidou et al., 2013). Variants included in the iCOGS project were prioritized for genotyping using the same approach as for the baseline ENIGMA variant list, with additional variants selected due to laboratory/bioinformatic evidence for effect on mRNA splicing. For each variant, a positive control DNA from a variant carrier was submitted for genotyping to facilitate calling of these rare variants. Lastly, we included a subset of variants for which multifactorial likelihood analysis results had previously been published, but the final classification reported was not “Class 5 Pathogenic” or “Class 1 Not Pathogenic”, and/or LRs were not all visible in the original publication (Easton et al., 2007; Farrugia et al., 2008; Lindor et al., 2012). Information from all these sources was collated for a total of 3,295 variants. This amalgamated list of variants was then circulated by email to ENIGMA consortium members to invite them to provide additional segregation or pathology information for inclusion in the analysis. Where relevant, additional nonoverlapping pathology or segregation information was sourced directly from publications for inclusion in the analysis. Overall, there were 1,008 informative pathology data points and 895 informative families for segregation analysis from ENIGMA collaborators, clinical enquiries, and nonoverlapping publications (see below for further explanation about LR assignment). After combining all information, at least one data point was available for 1,395 variants.

Variant descriptions are in accordance to HGVS recommendations. Nucleotide numbering corresponds to reference transcripts NM_007294.3 (*BRCA1*) and NM_000059.3 (*BRCA2*). Legacy description is also provided to assist comparison with historical records in the literature; the nucleotide numbering is from nucleotide one of the full gene sequence (Genbank: U14680.1/*BRCA1*; U43746/*BRCA2*) not the ATG initiator codon, and *BRCA1* exon boundaries are from GenBank U14680.1 with exon four missing due to a correction made after the initial description of the gene.

### 2.2 Multifactorial likelihood analysis

A Bayesian model was used to combine evidence as previously described (Goldgar et al., 2008). In brief, the prior probability of pathogenicity was assigned based on a combination of Align‐GVGD score and MaxEntScan splicing predictions, overlaid with expert knowledge incorporating prediction of variant effect on critical functional protein domains (Tavtigian et al., 2008; Vallee et al., 2016). Applicable prior probability of pathogenicity predictions for single nucleotide variants in *BRCA1* and *BRCA2* are available from the HCI Database of Prior Probabilities of Pathogenicity for Single Nucleotide Substitutions (http://priors.hci.utah.edu/PRIORS/). Align‐GVGD does not score in‐frame insertions and deletions, therefore to estimate prior probabilities for in‐frame deletion variants studied, we took the highest Align‐GVGD prior of the deleted bases. There were no in‐frame exonic insertions included in this study. The higher of the two priors (missense vs. splicing) was assigned for analysis. Co‐segregation analysis was performed as described by Thompson et al. (2003) for each family with more than one individual genotyped for the variant. Hazard ratio estimates were taken from Antoniou et al. (2003) for <30, 30–39, 40–49, 50–59, 60–69, 70–79, and 80+ age brackets. For individuals affected with ovarian cancer at 20–29 years, penetrance for ovarian cancer at age 30–39 was applied due to a lack of information in the younger penetrance class (Antoniou et al., 2003). If no age at last update was provided by the submitting center for unaffected individuals, test date was used to infer current age. Breast tumor pathology LRs were assigned based on the estimates in Spurdle et al. (2014), and considered age at diagnosis. When multiple tumors were present in one individual, the first diagnosed tumor with information available was taken, and only a single LR was assigned according to the extent of information available (out of the variables tumor grade, ER, PR, and HER2), following previous recommendations (Spurdle et al., 2014). Likelihood ratios for co‐occurrence with a pathogenic variant (*in trans*), and reported family history analysis, were drawn from a previous publication (Easton et al., 2007). Case‐control data from the iCOGS project were used to estimate LRs following methods described previously (de la Hoya et al., 2016).

Table S1 summarizes the LRs assigned for each component for each variant with at least one data point. Prior probabilities and LRs were combined to calculate posterior probabilities using Bayes rule: (Prior Probability x Combined LR)/(Prior Probability x [Combined LR + {1 − Prior Probability}]). Where multiple data points were available for a single data type, for example, segregation, LRs were combined multiplicatively. Using variant *BRCA1* c.131G>T as an example: Prior Probability is 0.81; Combined LR is 6,440.7 (based on LR Segregation (156.17) x LR Pathology (41.24) x LR Co‐occurrence (a) x LR Family History (a) x LR Case‐Control (a)). Posterior probability is 0.99996, calculated as (0.81 × 6440.7)/(0.81 × [6440.7 + {1‐0.81}]). Breakdown of clinical data type contributed, and the data sources (submitter, publication source), are shown in Table S2.

It has previously been proposed that a combined LR between 0.5 and 2, in particular if derived from a limited number of data points, provides insufficient observational data to perform a valid integrated analysis (Vallee et al., 2016). This is in accord with the idea that ACMG guidelines intrinsically include an indeterminate zone, between supporting benign and supporting pathogenic, for variants with insufficient or conflicting evidence for pathogenicity. Following this rationale, posterior probabilities of pathogenicity were not calculated for any variant with a combined LR between 0.5 and 2. Posterior probability of pathogenicity was calculated for a total of 734 variants, and classification assigned based on previously published cut‐offs proposed for the International Agency for Research into Cancer (IARC) five tier classification scheme (Plon et al., 2008), with some modification of terms used to describe tiers (Spurdle et al., 2019), namely: Class 5 Pathogenic, >0.99; Class 4 Likely Pathogenic, 0.95–0.99; Class 3 Uncertain, 0.05–0.949; Class 2 Likely Benign, 0.001–0.049; and Class 1 Benign, <0.001. The variant classifications, with breakdown of LR components and sources, have been submitted to the following databases for public display:

<http://hci‐exlovd.hci.utah.edu/home.php?select_db=BRCA1>

<http://hci‐exlovd.hci.utah.edu/home.php?select_db=BRCA2>

### 2.3 Datasets providing information for comparison and calibration of qualitative classification criteria

#### 2.3.1 ClinVar assertions

Variant pathogenicity assertions from ClinVar were taken from the November 2018 XML file. Summary annotation and individual submitter annotations are recorded in Table S1 (columns ClinVar Class Summary, ClinVar Class Details by Submitter). A small number of variants included had previously been reviewed by the ENIGMA *BRCA1/2* Expert Panel, but these have not been specifically annotated as the primary purpose of the comparison with ClinVar assertions was to identify discrepancies with classifications derived from this updated multifactorial likelihood analysis.

#### 2.3.2 mRNA splicing assay data

Published mRNA splicing assays of *BRCA1* and *BRCA2* variants were collated for a previous ENIGMA project (Walker et al., 2013), a resource which has been updated over time. Details of transcripts observed were recorded, and nomenclature errors for variant and transcripts corrected as necessary. We matched these results against the 1,395 variants included in this study (Table S1). Assays that provided variant allele‐specific transcript results from patient‐derived mRNA were noted as being eligible for assessment according to the ENIGMA classification guidelines, and effect on mRNA splicing was coded as none, partial, or complete (column Coded Splicing Effect). All other assay results (including additional assays of patient mRNA that did not measure allele‐specific expression, and construct‐based assays), were noted as to whether the variants were reported to impact mRNA splicing profile or not (column Allele‐Specific Splicing Result Summary). To simplify comparison with broad ACMG code description, the relationship of aberrant transcript/s to protein effect that is premature termination, disruption of clinically important residues was not captured in the mRNA effect codes. Further, to limit these LR measures to variant effect on mRNA splicing only, variants classified as (Likely) Pathogenic that had high bioinformatic prediction of effect on amino acid sequence were presumed to confer pathogenicity via effect on protein function, and excluded from the reference set for derivation of the mRNA splicing LRs. See Table S3 for details of the variants included in mRNA splicing subanalysis, and code assignments.

#### 2.3.3 Protein functional assay data

Seven relatively recent publications, providing results from different mammalian‐based assays of protein function, were chosen for comparison with classifications arising from multifactorial analysis. Five publications were from ENIGMA members and incorporate known pathogenic and known benign missense *BRCA1* or *BRCA2* variants as controls, permitting estimation of sensitivity, and specificity of missense variant pathogenicity: (a) region‐limited construct‐based transcriptional activation assays of missense variants located across the *BRCA1* Coiled Coil, BRCT1, Linker, and BRCT2 domains (Fernandes et al., 2019); (b) full‐length complementary DNA (cDNA) construct‐based homologous recombination DNA repair assays of missense variants in the *BRCA2* C‐terminal DNA binding domain (Hart et al., 2019); (c) a mouse embryonic stem cell‐based assay of *BRCA1* variants assessing the ability of full‐length human *BRCA1* cDNA to complement reduced cell proliferation following deletion of a conditional mouse *Brca1* allele, and cisplatin sensitivity (Bouwman et al., 2013); (d) a mouse embryonic stem cell‐based assay of *BRCA2* variants assessing the ability of full‐length genomic human *BRCA2* to complement loss of cell viability following deletion of a conditional mouse *Brca2* allele, and follow‐on homologous recombination assays of *BRCA2*‐expressing cells that are able to complement cell lethality (Mesman et al., 2019); and (e) analysis of homologous recombination for *BRCA1* BRCT missense variants expressed in mammalian cells as *BRCT1*–*BRCT2* clones into pcDNA3 (modified)‐full‐length *Brca1*, followed by measures of human *BRCA1 BRCT1*–*BRCA2* domain solubility (assayed in bacterial cells), and phosphopeptide‐binding properties of the purified BRCT domain variants in vitro (Petitalot et al., 2019). These selected studies were the most recent publications arising from these research groups, and incorporated research results from any prior publications from that group. Two additional publications were selected as they provide results from high‐throughput multiplex assays. The first was a multiplex reporter assay characterizing the effects of 1,056 amino acid substitutions in the first 192 residues of *BRCA1* on homology‐directed repair of double strand breaks (Starita et al., 2018). The second was a high‐throughput saturation genome editing haploid cell survival assay, measuring functional effect for single nucleotide variants in 13 *BRCA1* exons covering key functional domains (Findlay et al., 2018): targeted genomic DNA sequencing and RNA sequencing were used to quantify variant abundance and infer effect on *BRCA1* function overall (DNA sequencing), and via effect on mRNA production (RNA sequencing); function scores for different exons were normalized by matching median scores for synonymous and nonsense variants to global medians for these variant types. The assay design for five of the seven studies permitted assay of effect on protein only, and not mRNA levels or splicing. We thus excluded “missense” variants that were known to alter mRNA levels or splicing, or were located in the splicing motifs at the start/end of exons, such that comparisons with results from functional studies should all be limited to measures of variant effect on protein only. Effect on function as assigned by the original publication was recoded into one of three tiers (complete, partial, or no functional impact). A total of 77 unique variants assayed by at least one study were available for comparison with multifactorial likelihood classifications. See Table S4 for details of the variants and functional code assignments. For Mesman et al. (2019), results from complementation and homologous recombination assay combined were used to assign final functional effect as per recommendations from those authors. For Petitalot et al. (2019), functional classification was based on a combination of homologous recombination, BRCT solubility, and phosphopeptide binding (from their own study and also reports in the literature), as detailed in Table 1b of the publication (Petitalot et al., 2019).

#### 2.3.4 Reference population frequency data

Population frequency data were downloaded from the Genome Aggregation Database (gnomAD non‐cancer v2.1 dataset; gnomad.broadinstitute.org). Three large in‐frame deletion variants classified as Pathogenic were excluded from the population frequency LR estimation analysis, as frequencies are not provided by gnomAD for variants of this type. No large deletion variants were classified as (Likely) Benign. As an exercise to compare the validity of gnomAD as a “control” reference dataset, we also compared the frequency of 17 variants detected at allele frequency <1% in European ancestry breast cancer‐free controls (*n* = 38,694) genotyped as part of iCOGS, to frequencies for the same variants observed in (a) non‐Finnish Europeans from gnomAD (maximum *n* = 59,073, from genome and exome data combined), and (b) European aged (>70 year old) cancer‐free control from the FLOSSIES dataset (*n* = 7,325; https://whi.color.com/). Our comparisons of variant frequency in gnomAD to those of “true” controls of European ancestry (Table S5) indicated that variants were more likely to be absent from the smaller FLOSSIES dataset (only 6/17 variants were observed), whereas the frequency category based on the gnomAD non‐Finnish Europeans was the same as that for iCOGS European ancestry controls for all 17 variants. The FLOSSIES African dataset provides allele frequencies based on 2,559 individuals (5,118 alleles), which is considerably smaller than the gnomAD African dataset. For this reason we opted to use only gnomAD outbred (non‐founder) sample sets (non‐Finnish European [15,316–118,174], African [8,664–23,620], Latino [22,398–35,108], South Asian [21,600–30,526], East Asian [14,012–19,252]) to determine the highest minor allele frequency observed based on exome and genome data combined. Variants observed only once across all five outbred sample sets were annotated as such for frequency LR estimation (Table S1). Variants absent from all five outbred sample sets, but present once in Finnish or Ashkenazi Jewish sample sets were excluded from the frequency LR estimation (noted as “NA” in column “Frequency Category Assigned for LR derivation” in Table S1).

Designation of LRs to ACMG/AMP rule code strengths were based on LR ranges recently proposed as consistent with ACMG/AMP qualitative rule strengths for future classification in a Bayesian framework (Tavtigian et al., 2018). Namely: supporting evidence for pathogenicity, LR 2.08–4.3; moderate evidence for pathogenicity, LR 4.3–18.7; and strong evidence for pathogenicity; LR 18.7–350. LRs ranges for Benign code strengths were calculated as the inverse of the ranges proposed for Pathogenic code strengths.

## 3 RESULTS AND DISCUSSION

An IARC class informative for clinical management (Class 1 Benign, Class 2 Likely Benign, Class 4 Likely Pathogenic, or Class 5 Pathogenic) was reached for 541/734 (74%) of the 734 variants considered to have sufficient information to inform calculation of a posterior probability of pathogenicity (Table S1). The remaining 193/734 variants fell within the wide range of 0.05–0.95 considered inconclusive of variant pathogenicity (Class 3 Uncertain). Overall, only 54 of the 541 variants with posterior probability assigned have previously been assessed using the multifactorial likelihood approach, and in some instances the clinical evidence included previously did not pass thresholds that have since been set for combined LR (between 0.5 and 2) considered informative for quantitative analysis (see Methods). Of the 541 variants falling outside of Class 3 Uncertain, 67 (9% of the 734 assessed) had a posterior probability of pathogenicity > 0.99 (Class 5 Pathogenic), and 27 (4%) had a posterior probability of pathogenicity > 0.95 (Class 4 Likely Pathogenic), classifications which directly influence management of variant carriers and their relatives (Table 1), with 447 (61%) classified as (Likely) Benign. These findings are consistent with the knowledge that the majority of missense and intronic variants in these genes will, a priori, not be associated with a high risk of cancer as they fall outside of or are unlikely to impact BRCA1 or BRCA2 protein functional domains.

**Table 1:** Variants classified as Pathogenic or Likely Pathogenic using multifactorial likelihood analysis^a^

Gene	HGVS nucleotide	HGVS protein	Prior probability of pathogenicity	Co‐segregation LR	Tumor pathology LR	Co‐occurrence LR	Family history LR	Combined LR	Posterior probability of pathogenicity	IARC 5‐tier class
*BRCA1*	c.131G>T	p.(Cys44Phe)	0.81	156.17	41.24			6,440.72	1.000	P
*BRCA1*	c.135‐1G>T		0.97	9,528.00	2.01			19,140.38	1.000	P
*BRCA1*	c.140G>A	p.(Cys47Tyr)	0.81	13,545.00	60.31			816,898.95	1.000	P
*BRCA1*	c.191G>A	p.(Cys64Tyr)	0.81	5,300,394.00	189.09			1,002,254,925.72	1.000	P
*BRCA1*	c.211A>G	p.(Arg71Gly)	0.81	383.27				383.27	0.999	P
*BRCA1*	c.212+1G>A		0.97	1,794.18	3.73			6,692.28	1.000	P
*BRCA1*	c.212+1G>T		0.97	7.65	3.34			25.56	0.999	P
*BRCA1*	c.213‐12A>G		0.97	29,429.38	1,132.14			33,318,129.21	1.000	P
*BRCA1*	c.302–2del		0.97	58,263.44	387.67	1.43	1.87	60,436,990.13	1.000	P
*BRCA1*	c.3G>T	p.(Met1?)	0.96	1.97	3.73			7.36	0.994	P
*BRCA1*	c.4097‐2A>G		0.97	0.42	9.99			4.20	0.993	P
*BRCA1*	c.4185+2_4185+22delinsA		0.96	0.33	30.65	1.26	28.61	358.47	1.000	P
*BRCA1*	c.4185G>A	p.(=)	0.97	39.32	13.91			547.01	1.000	P
*BRCA1*	c.4357+1G>T		0.97	0.67		1.14	6.63	5.10	0.994	P
*BRCA1*	c.4484+1del		0.97	0.33	28.14			9.22	0.997	P
*BRCA1*	c.4484G>C	p.(Arg1495Thr)	0.34	27.73	9.99			276.94	0.993	P
*BRCA1*	c.4485‐1G>T		0.97			1.03	11.76	12.15	0.997	P
*BRCA1*	c.4675+3A>T		0.97			1.03	4.18	4.31	0.993	P
*BRCA1*	c.4675G>A	p.(Glu1559Lys)	0.97	1.79		1.26	3.48	7.84	0.996	P
*BRCA1*	c.4676‐1G>A		0.97	3.75		2.92	32.31	353.30	1.000	P
*BRCA1*	c.4986+6T>G		0.97	18.94	1,134.07			21,484.60	1.000	P
*BRCA1*	c.4987‐2A>G		0.97	1.99	9.70	1.05		20.20	0.998	P
*BRCA1*	c.5062_5064del	p.(Val1688del)	0.66	16.93		1.18	13.41	267.23	0.998	P
*BRCA1*	c.5074+1G>T		0.97	13.92	1.69	1.10	1.18	30.64	0.999	P
*BRCA1*	c.5074+2T>C		0.97	2.43	3.73	1.07	7.31	70.75	1.000	P
*BRCA1*	c.5074G>C	p.(Asp1692His)	0.97	1.91	3.73	1.25	0.90	8.02	0.996	P
*BRCA1*	c.5089T>C	p.(Cys1697Arg)	0.81	730.63	2.41			1,758.02	1.000	P
*BRCA1*	c.5144G>A	p.(Ser1715Asn)	0.66	57.86	3.73	1.30	3.63	1,016.90	0.999	P
*BRCA1*	c.5153‐1G>A		0.97			1.22	3.11	3.78	0.992	P
*BRCA1*	c.5277+1_5277+6del		0.97			1.03	15.10	15.60	0.998	P
*BRCA1*	c.53T>C	p.(Met18Thr)	0.66	1.84	5.28	1.22	25.48	300.74	0.998	P
*BRCA1*	c.5468‐1G>A		0.97	2.10	41.24	1.03	1.24	110.47	1.000	P
*BRCA1*	c.547+1G>T		0.97	1.95	1.67			3.25	0.991	P
*BRCA1*	c.5509T>C	p.(Trp1837Arg)	0.81	183.54	0.73	1.14	0.19	28.33	0.992	P
*BRCA1*	c.5516T>C	p.(Leu1839Ser)	0.66	21.96	51.90			1,139.69	1.000	P
*BRCA1*	c.81‐9C>G		0.97			1.07	8.13	8.67	0.996	P
*BRCA2*	c.(?_‐227)_67+?del	p.?	0.96	13.34	1.17			15.56	0.997	P
*BRCA2*	c.3G>A	p.(Met1?)	0.96	67.62	2.17	1.07		157.49	1.000	P
*BRCA2*	c.475+1G>T		0.97	1.90		1.21	9.19	21.21	0.999	P
*BRCA2*	c.517‐2A>G		0.97	3.50	3.82	1.28	3.12	53.08	0.999	P
*BRCA2*	c.631+2T>G		0.97	22.51	0.81			18.28	0.998	P
*BRCA2*	c.632‐1G>A		0.97	95.33	2.21			210.58	1.000	P
*BRCA2*	c.632‐2A>G		0.97	152.33	0.79	1.05	0.18	23.32	0.999	P
*BRCA2*	c.632‐3C>G		0.97			1.05	3.60	3.78	0.992	P
*BRCA2*	c.67+1G>T		0.97	3.91	1.19	1.05	1.48	7.21	0.996	P
*BRCA2*	c.6938‐2A>G		0.97			1.08	5.30	5.70	0.995	P
*BRCA2*	c.7007G>A	p.(Arg2336His)	0.34	48.57	0.83	2.46	20.33	2,013.98	0.999	P
*BRCA2*	c.7008‐?_7805+?del		0.81	2,786.04	18.12			50,481.75	1.000	P
*BRCA2*	c.7008‐1G>A	p.(=)	0.97	2.00	1.79			3.57	0.991	P
*BRCA2*	c.7008‐2A>G		0.97	1.52		1.02	2.50	3.90	0.992	P
*BRCA2*	c.7617+2T>G		0.97	2.72	0.81	1.08	1.59	3.75	0.992	P
*BRCA2*	c.7618‐2A>G		0.97	1.30	1.96	1.03	2.29	6.03	0.995	P
*BRCA2*	c.7806‐2_7806–1dup		0.99	8.77	4.38			38.36	1.000	P
*BRCA2*	c.7806‐2A>G		0.97	7.11	1.20	1.41	1.67	20.04	0.998	P
*BRCA2*	c.7975A>G	p.(Arg2659Gly)	0.81	1,950.00	0.86	1.10	5.07	9,380.28	1.000	P
*BRCA2*	c.7976+1G>A		0.97	0.71		1.22	160.57	138.22	1.000	P
*BRCA2*	c.7977‐1G>C		0.97	1,984,739.68	10.20			20,246,907.80	1.000	P
*BRCA2*	c.8023A>G	p.(Ile2675Val)	0.64	605.14	0.88	1.05	2.88	1,612.05	1.000	P
*BRCA2*	c.8488‐1G>A		0.97			1.13	3.73	4.21	0.993	P
*BRCA2*	c.8632+1G>A		0.97	21.93	0.74			16.19	0.998	P
*BRCA2*	c.8633‐24_8634del		0.97			1.05	3.16	3.32	0.991	P
*BRCA2*	c.8633‐2A>G		0.97	1.29	1.06	1.05	6.65	9.58	0.997	P
*BRCA2*	c.8755‐1G>A		0.97	9.40	2.85	1.31	8.13	284.48	1.000	P
*BRCA2*	c.8954‐1_8955delinsAA		0.97			1.28	3.77	4.81	0.994	P
*BRCA2*	c.9118‐2A>G		0.97	10.89	0.51			5.56	0.994	P
*BRCA2*	c.9257‐?_(*1_?)del		0.81	31.48	3.10			97.53	0.998	P
*BRCA2*	c.9371A>T	p.(Asn3124Ile)	0.81	402,919.02	0.54	2.07	81.64	36,698,863.68	1.000	P
*BRCA1*	c.181T>A	p.(Cys61Ser)	0.81	1.39	3.73			5.19	0.957	LP
*BRCA1*	c.1A>G	p.(Met1?)	0.96	1.27	2.23			2.83	0.985	LP
*BRCA1*	c.230_238del	p.(Thr77_Phe79del)	0.66	1.83	16.45			30.16	0.983	LP
*BRCA1*	c.4185_4185+3del		0.97	2.09		1.048		2.19032	0.986	LP
*BRCA1*	c.4185+1G>T		0.97			1.10	2.64	2.91	0.989	LP
*BRCA1*	c.4479_4484+2dup		0.97	0.96	3.16			3.04	0.990	LP
*BRCA1*	c.4676‐2A>G		0.97			1.03	2.29	2.36	0.987	LP
*BRCA1*	c.5193+1G>A		0.97	1.27	1.67			2.12	0.986	LP
*BRCA1*	c.5207T>G	p.(Val1736Gly)	0.81			1.10	6.53	7.20	0.968	LP
*BRCA1*	c.5213G>A	p.(Gly1738Glu)	0.81	1.18		1.18	8.89	12.30	0.981	LP
*BRCA1*	c.5216A>T	p.(Asp1739Val)	0.81	0.82		1.03	7.93	6.72	0.966	LP
*BRCA1*	c.5243G>A	p.(Gly1748Asp)	0.81	3.80	3.16	1.03	0.92	11.47	0.980	LP
*BRCA1*	c.5333‐6T>G		0.97	0.68	3.73			2.55	0.988	LP
*BRCA1*	c.5558A>G	p.(Tyr1853Cys)	0.81	12.66	0.40	1.07	2.29	12.39	0.981	LP
*BRCA1*	c.80+5G>A		0.34	15.05	1.49	1.03	2.60	60.32	0.969	LP
*BRCA2*	c.476‐1G>A		0.97	1.23		1.02	2.18	2.75	0.989	LP
*BRCA2*	c.67+2T>C		0.97			1.13	1.86	2.10	0.985	LP
*BRCA2*	c.681+1G>A		0.97	1.94	1.08			2.10	0.985	LP
*BRCA2*	c.7007+5G>C		0.34	8.55	0.95	1.08	4.75	41.64	0.955	LP
*BRCA2*	c.7819A>C	p.(Thr2607Pro)	0.66			1.02	11.62	11.90	0.959	LP
*BRCA2*	c.8009C>T	p.(Ser2670Leu)	0.29	17.87	4.74	1.37	1.22	141.76	0.983	LP
*BRCA2*	c.8035G>T	p.(Asp2679Tyr)	0.81	3.54	1.06	1.02	1.24	4.76	0.953	LP
*BRCA2*	c.8331+1G>A		0.97			1.02	2.72	2.79	0.989	LP
*BRCA2*	c.8331+1G>T		0.97	1.17	1.77			2.07	0.985	LP
*BRCA2*	c.8754+4A>G		0.34	4.87	3.13	1.34	2.22	45.36	0.959	LP
*BRCA2*	c.8975_9100del	p.(Pro2992_Thr3033del)	0.81			1.24	13.48	16.78	0.986	LP
*BRCA2*	c.9257‐1G>C		0.34	1.29	0.76	1.16	47.50	54.24	0.965	LP

Abbreviations: HGVS, Human Genome Variation Society; LP, Likely Pathogenic; LR, likelihood ratio; P, Pathogenic.

^a^ See methods for overview of sources of information, and multifactorial likelihood analysis methods. Case‐control LR data were not available for any variants classified as (Likely) Pathogenic.

Further, we draw attention in particular to two variants (*BRCA2* c.516+1G>T and *BRCA2* c.7007+1G>C) demonstrating differences between multifactorial likelihood‐based analysis and the current iteration of ENIGMA “rules‐based” qualitative assessment based on mRNA splicing assay data from patient material (Houdayer et al., 2012; Whiley et al., 2011). The splicing assay data, albeit not allele‐specific, indicate that both variants impact splicing profile. According to ENIGMA qualitative classification criteria, the *BRCA2* c.516+1G>T (intron 6) and *BRCA2* c.7007+1G>C (intron 13) variants would be classified as Class 4 Likely Pathogenic based on their location in a donor dinucleotide ‐ in the absence of conflicting information. Despite the high prior probability of 0.97 based on bioinformatic prediction, the clinical information included in this study provided sufficient evidence against pathogenicity that the posterior probability fell below 0.95 (0.81 for *BRCA2* c.516+1G>T, 0.78 for *BRCA2* c.7007+1G>C). For *BRCA2* c.7007+1G>C, the variant was identified in the breast cancer affected proband but not in the one affected relative tested. For *BRCA2* c.516+1G>T, only two of four affected relatives tested were carriers. Although both variants have been submitted to ClinVar as pathogenic by multiple submitters; summary evidence was provided for only one assertion for *BRCA2* c.7007+1G>C, and refers to variant location and splicing assay data with no additional clinical details. Interestingly, unpublished results from mouse embryonic stem cell assays M.P.G. Vreeswijk (personal communication, 22 January 2019) indicate that *BRCA2* c.7007+1G>C has a severe impact on function as measured by failure to complement the lethal cell phenotype, whereas *BRCA2* c.516+1G>T does not have a severe impact on function (complementation; 56% HDR capacity, within the range for variants previously placed in Class 1/2 by multifactorial likelihood analysis). The combined observations for these two variants raise the complex issue of what constitutes sufficient conflicting information when assigning a qualitative classification, or perhaps even in the context of LRs included in a quantitative classification calculation. The current classification in ClinVar as Likely Pathogenic would appear to be consistent with ACMG‐derived classifications used in clinical practice, but we strongly recommend prioritized collection of additional clinical, splicing and functional data to provide more extensive information in support of assertions for these two variants.

Overall, comparison of classes assigned by multifactorial likelihood analyses and pathogenicity assertions in ClinVar revealed that of the 94 variants classified as (Likely) Pathogenic by multifactorial likelihood analysis conducted in this study, 80 have at least one assertion as (Likely) Pathogenic, seven are Uncertain, and seven are currently not in ClinVar; that is none were submitted as (Likely) Benign. Of the 447 variants classified as (Likely) Benign using multifactorial analysis, 212 have at least one assertion as (Likely) Benign, 234 were either uncertain in or absent from ClinVar, and the remaining variant *BRCA1* c.5453A>G p.(Asp1818Gly) is actually a spliceogenic variant with four assertions as (Likely) Pathogenic. The explanation for this discrepancy is detailed below. Altogether, these results can now be used to contribute to ENIGMA expert panel classification of 541 variants, 248 of which are new or considerably altered compared to current submissions to ClinVar.

### 3.1 Correlation of multifactorial likelihood classifications with splicing assay data

Current ENIGMA *BRCA1/2* classification criteria for spliceogenic variants, consistent with those of the InSiGHT Consortium developed for classification of mismatch repair gene variants (Thompson et al., 2014), present stringent recommendations for use of mRNA splicing data for variant interpretation (https://enigmaconsortium.org). Namely, a variant is only considered pathogenic on the basis on mRNA splicing data if there is no predicted functional transcript produced from the variant allele, as determined by assays of patient‐derived mRNA that have assessed allele‐specific expression of alternate transcripts. This stipulation is not specified for ACMG/AMP classification codes using splicing data (PS3, well‐established in vitro or in vivo functional studies supportive of a damaging effect on the gene or gene product).

We undertook a comparison of multifactorial model classifications against published splicing assays results (including assays of patient material and construct‐based assays) to calibrate use of splicing assay data for use as weighted information for qualitative classification, based on the LR ranges recently proposed as consistent with ACMG/AMP qualitative rule strengths for future classification in a Bayesian framework (Tavtigian et al., 2018). Of the variants falling outside of Class 3 Uncertain in this analysis, 99 had mRNA splicing data available, 25 of which had been assessed using allele‐specific assays of mRNA from patient tissue. By comparing splicing effect to classifications derived from this study, we estimated a LR towards pathogenicity based on effect on mRNA splicing (Table 2; Table S3 for additional details). The very limited number of allele‐specific assays did not allow for robust estimates of LRs, with the confidence intervals for LRs estimated for both partial and complete effect on splicing including unity. Nevertheless, results support the hypothesis that partial effect on splicing will not be as strongly predictive of pathogenicity as is complete effect on splicing (LR 3.82 vs. LR 6.36 from this analysis). Including all assay results, no effect on splicing provided strong evidence against pathogenicity (LR 0.02), whereas any impact on splicing (without measurement of allele‐specific effects, or consideration of in‐frame transcripts) provided moderate evidence for pathogenicity (LR 12.24). Recognizing the small sample sizes, and consequently large confidence limits, these results nevertheless demonstrate the value of mRNA splicing assays as a component in qualitative variant classification. We also highlight the possibility that there is likely to be considerable bias in variants selected for mRNA assays, with over‐representation of variants at the highly conserved donor and acceptor dinucleotides, positions that, when altered, are likely to impact splicing more severely than spliceogenic variants located at other positions. We thus stress the importance of incorporating allele‐specific expression assays into variant evaluation processes wherever possible, and to revisit such analysis with larger datasets in the future. We also recommend that future larger‐scale comparisons to derive LRs for splicing assay data should consider in greater detail the predicted impact of the aberrant mRNA profiles on protein function, and in particular consider the relevance of in‐frame isoforms that could be translated to result in (partially) functional protein. Further, as both partial mRNA splicing and in‐frame transcripts may be associated with reduced cancer penetrance that is not inherently captured by the design of the multifactorial model, family‐based and case‐control studies may be necessary to tease out which such spliceogenic variants are indeed risk‐associated, and if this level of risk is clinically actionable.

**Table 2:** Splicing effect reported for variants classified as (Likely) Benign or (Likely) Pathogenic using multifactorial likelihood analysis^a^

Splicing effect	(Likely) benign		(Likely) pathogenic		LR towards pathogenicity	(95% confidence interval)
	n	%	n	%		
***Assays measuring allele‐specific expression***						
None	11	78.57	0	9.09^b^	0.12	(0.02–0.76)
Partial	2	14.29	6	54.55	3.82	(0.95–15.36)
Complete	1	7.14	5	45.45	6.36	(0.86–46.86)
*Total*	*14*		*11*			
***All splicing results***						
None	46	92.00	0	2.04^b^	0.02	(0.01–0.15)
Any impact	4	8.00	49	97.96	12.24	(4.78–31.35)
*Total*	*50*		*49*			

^a^ See methods for overview of sources of mRNA splicing information, and categorization of splicing effect. Also see Table S3 for details of variants included in comparison.

^b^ Percentage is calculated assuming a single variant in this category, and thus provides a conservative estimate of the LR.

We then considered qualitative classification based on mRNA splicing results for all variants with a multifactorial likelihood calculation that is including Class 3 Uncertain variants (See Table S1, columns Splicing Results/s and Allele‐Specific Assay). Following ENIGMA *BRCA1/2* qualitative classification criteria (http://enigmaconsortium.org/), there were 15 variants that could be interpreted as Pathogenic based on splicing, that is no predicted functional transcript produced from the variant allele; of these, multifactorial data classified five as (Likely) Pathogenic, five as Uncertain, whereas four had insufficient data to perform a calculation. *BRCA1* c.5453A>G was the only variant with truly discordant classification between splicing results (Class 5 Pathogenic) and multifactorial data analysis (Class 2 Likely Benign); the multifactorial classification was based on low prior probability of 0.03 for the presumed missense substitution Asp1818Gly, one pathology data point (LR 0.34), and relatively uninformative co‐occurrence (LR 1.12) and family history (LR 0.91) data. This variant highlights a recognized limitation of current bioinformatic predictions used in the multifactorial analysis; the variant alters splicing by modifying an exonic splice enhancer (ESE; Rouleau et al., 2010). There are currently no bioinformatic prediction tools with adequate sensitivity and specificity to predict ESE loss or gain with any reliability, and this mechanism has thus not yet been incorporated into bioinformatic prior probability estimation. Although results from splicing assays can obviously add value for such examples, the poor predictability of ESEs and effects of variation on ESE function, hinders the prioritization of ESE‐altering variants for splicing assays. We reiterate that the Class 2 Likely Benign tier implicitly allows for a 5% error rate in classification, and resources permitting, we would encourage additional data collection for all variants falling in this tier. Moreover, future inclusion of a LR derived for splicing impact as a component of the multifactorial likelihood analysis, where such splicing information is available, would likely shift the posterior probability for such variants into the range of Class 3 Uncertain and so prevent overt misclassification driven by bioinformatic prediction deficiencies. At this point in time, we would encourage additional clinical data collection for *BRCA1* c.5453A>G to confirm that the clinical phenotype is consistent with a Class 5 Pathogenic assertion based on splicing data only.

### 3.2 Correlation of multifactorial likelihood classifications with protein functional assay data

Functional assays are considered strong evidence for or against pathogenicity using ACMG/AMP codes PS3 and BS3 (well‐established in vitro or in vivo functional studies show (damaging/no damaging) effect on gene or gene product). A range of different assays have been used to assess effect of *BRCA1* and *BRCA2* variants on protein function, some limited to measuring impact on function of variants within a specific domain, and others measuring output relevant to a variant located anywhere in the coding region. To assess the strength of this evidence as a predictor of the clinical significance of anticipated missense *BRCA1* and *BRCA2* variations, it is important to consider several factors. Sensitivity and specificity of assays should be determined using missense variants that have previously been determined to be pathogenic or benign (Guidugli et al., 2014; Millot et al., 2012); that is assay profiles for truncating variants may not be appropriate to measure loss of function displayed by pathogenic missense variants. To prevent circularity, functional assay results should not have contributed to the classification of these “control” missense variants, as may be the situation for variants submitted to ClinVar as pathogenic. An additional factor to consider, but not addressable at this point in time, is that there are few *BRCA1/2* variants robustly proven to be associated with moderate risk of cancer. There is thus a paucity of controls to calibrate assay results to detect moderate‐risk variants. Moderate‐risk variants are intuitively expected to have less impact on function than variants associated with a high cancer risk comparable to that of the average truncating allele, and severity of their impact on function may differ depending on the specific protein effects measured (Lovelock et al., 2007).

For this reason, we compared our multifactorial analysis results for missense variants classified outside of Class 3 Uncertain to results from selected published functional assays (also see Methods). Briefly, these included: (a) domain‐specific or generic assays assessing variant effect on protein function, and calibrated against missense variants previously classified as pathogenic or benign using multifactorial likelihood analysis that is using bioinformatic and clinical information (Bouwman et al., 2013; Fernandes et al., 2019; Hart et al., 2019; Mesman et al., 2019; Petitalot et al., 2019); and (b) multiplex reporter assays (Findlay et al., 2018; Starita et al., 2018) reported to have reasonable to good sensitivity and specificity by comparison to ClinVar classifications (including truncating, splicing, and missense variants). There were 16 (Likely) Pathogenic and 61 (Likely) Benign variants with a protein functional assay result from at least one study (Table 3; Table S4 for additional details). All 56 variants reported to have no functional impact were classified as (Likely) Benign, as were the four of five variants demonstrating partial function in at least one assay. The fifth variant *BRCA1* c.5216A>T p.(Asp1739Val) was classified as Likely Pathogenic based on posterior probability of 0.97. As outlined in Supp Table S4, this missense substitution variant was reported to have complete loss of function using transcription activation and cell survival assays, but partial activity by Petitalot et al. (2019); the latter categorization was based on the combination of somewhat decreased solubility, decreased BACH1 binding (reported in yet another publication, Lee et al. (2010)), and normal homologous recombination and localization (Petitalot et al., 2019). Of the 16 variants reported to impact function completely (and with no evidence otherwise by another of the functional studies selected), 15 were classified by multifactorial likelihood analysis as (Likely) Pathogenic, and the other as (Likely) Benign. Of note, the latter variant *BRCA2* c.8351G>A p.(Arg2784Gln) did complement lethality in the mouse embryonic stem cell assay (Mesman et al., 2019), but was coded as impacting function based on homologous recombination assay results from the same study (Mesman et al., 2019), and was reported to impact homologous recombination in an independent study (Hart et al., 2019). We note that for the two exceptions highlighted (*BRCA1* c.5216A>T, *BRCA2* c.8351G>A), the results from survival assays were concordant with the multifactorial likelihood classification.

**Table 3:** Functional effect reported for missense substitution variants classified as (Likely) Benign or (Likely) Pathogenic using multifactorial likelihood analysis^a^

Protein functional effect in at least 1 study^b^	(Likely) benign		(Likely) pathogenic		LR towards pathogenicity	(95% confidence interval)
	n	%	n	%		
None	56	91.80	0	6.25^c^	0.07	(0.01–0.45)
None/partial	3	4.92	0		N/A	
Partial	1	1.64	0		N/A	
Partial/complete	0		1	6.25	N/A	
Complete	1	1.64	15	93.75	57.19	(8.15–401.14)
*Total*	*61*		*16*			

^a^ Excludes missense variants shown to be associated with altered mRNA splicing, or with reduced/absent mRNA expression from survival assays. See Table S4 for details of variants included in comparison.

^b^ Functional impact codes assigned based on effect description as originally published. See Table S4 for more details.

^c^ Percentage is calculated assuming a single variant in this category, and thus provides a conservative estimate of the LR.

Considering results overall, we estimated a LR towards pathogenicity based on assays of protein function from at least one study (Table 3). Acknowledging the caveat of small sample sizes, and at least one observation (and thus liberal frequency estimates) assumed for cells without counts, our results support use of functional assay data as moderate or strong evidence in determining pathogenicity assertions for missense variants. Specifically, complete impact on function with no conflicting evidence is strongly predictive of missense variant pathogenicity (estimated LR 57.19, lower confidence bound 8.15). No impact on protein function provides moderate evidence against missense variant pathogenicity (LR 0.07 with upper bound 0.45, equating to an LR of 15.26 against pathogenicity). The results confirm the value of results from these selected protein functional assays as a component in qualitative classification of missense variants. They also stress the importance of considering discordances across different assay methods as an approach to select individual variants for further consideration as potential moderate‐risk variants, variants that may not always be detectable as risk‐associated using statistical models developed for high‐risk variants. Further, as noted before, the *BRCA1/2* multifactorial model is designed to capture clinical features of patients with the average high‐risk pathogenic variant, and we cannot exclude the possibility that some variants demonstrating impact on function (partial, or even complete for at least one assay type) are moderate‐risk alleles. It will thus be important to prioritize variants such as *BRCA1* c.5216A>T p.(Asp1739Val) and *BRCA2* c.8351G>A p.(Arg2784Gln), where some functional data conflict the clinical information data (thereby arguably considered Uncertain according to ACMG/AMP qualitative criteria) for further study as potential moderate‐risk variants.

### 3.3 Correlation of multifactorial likelihood classifications with frequency in reference population datasets

Variant frequency in disease‐free controls can be used to provide evidence against pathogenicity, and indeed minor allele frequency (MAF) > 1% in a nonfounder population is considered stand‐alone evidence against pathogenicity for *BRCA1/2* variants by the ENIGMA consortium. An algorithm to define a “maximum credible population allele frequency” (Whiffin et al., 2017) has been proposed as a method to select MAF cut‐offs as evidence against pathogenicity, and indeed was used as a basis to select relevant minor allele frequency cut‐offs for the *PTEN* and *CDH1* adaptations of ACMG/AMP rule codes BA1 (stand‐alone) and BS1 (strong) evidence against pathogenicity, described as “allele frequency is greater than expected for the disorder.” The output of this algorithm can vary widely depending on input assumptions for disease penetrance and prevalence of the disorder, and is complicated for multicancer syndromes where penetrance varies for cancer type and even cancer subtype. Further, absence from control datasets has been proposed as moderate evidence for variant pathogenicity (ACMG/AMP rule code PM2).

The most commonly used “control” reference sets (ExAC, and more recently gnomAD) include males and females that were ascertained for noncancer related studies mostly at ages younger than the average age at onset of *BRCA1/2*‐related breast or ovarian cancer, but individual‐level information about cancer phenotypes is not available. Even assuming that these reference sets are largely cancer unaffected, it must be considered that penetrance in female pathogenic variant carriers for breast cancer is not complete, and much lower for male carriers. Indeed, known pathogenic *BRCA1/2* variants have been identified in these population control sets, even after accounting for “founder” pathogenic variants (Maxwell, Domchek, Nathanson, & Robson, 2016). As a result, the current ENIGMA *BRCA1/2* classification guidelines used empirical data to select frequency cut‐offs for qualitative classification criteria (https://enigmaconsortium.org/). Specifically, allele frequency ≥ 0.001 and < 0.01 in large outbred control reference groups was selected as a component of evidence against pathogenicity, based on the upper 95% confidence interval (binomial Exact) of the frequency observed for the most common pathogenic allele in non‐Finnish European and other population groups drawn from ExAC and gnomAD. The absence from controls has not yet been incorporated into the ENIGMA *BRCA1/2* guidelines.

To determine the utility of variant frequency in (or absence from) reference population sets for future *BRCA1* and *BRCA2* variant classification, and to formally assess the strength of this evidence, we estimated a LR based on MAF in gnomAD v2.1 (noncancer), and considered them against LR cut‐offs suggested for ACMG/AMP rules (Tavtigian et al., 2018). Results are shown in Table 4. We considered variants observed only once across all sample sets reviewed as a separate category (see Methods), and categorized the remaining variants into three MAF bins: 0.01 > MAF ≥ 0.0001; 0 < MAF < 0.0001; and not observed. The proportion of variants seen only once across all five sample sets was 13.32% for (Likely) Benign variants compared with 8.99% for (Likely) Pathogenic Variants, which equates to an LR of 0.67, considered uninformative for pathogenicity prediction. Variants classified as (Likely) Benign were spread relatively evenly across the frequency categories, whereas (Likely) Pathogenic variants were only seen at MAF < 0.0001, and the vast majority (88%) were not seen in population controls. Assuming conservatively a single (Likely) Pathogenic variant to fall in the category “ ≥ 0.0001 & < 0.01,” the LR estimate is 0.05, equating to an LR of 22.10 (3.12–156.21) against pathogenicity, considered strong evidence that a variant is benign. The estimated LR against pathogenicity for a variant seen in gnomAD at MAF < 0.0001 is 7.97 (2.59–24.50), which meets moderate evidence against pathogenicity. Last, the estimated LR towards pathogenicity for a variant not detected in gnomAD is 2.50 (2.16–2.91), corresponding to supporting evidence in favor of pathogenicity; these findings suggest that whereas “absence in controls” may be useful for *BRCA1/2* variant classification, such evidence should carry less weight than the PM2 moderate code proposed by ACMG/AMP for generic use.

**Table 4:** Frequency in reference population control sets for variants classified as (Likely) Benign or (Likely) Pathogenic using multifactorial likelihood analysis^a^

	(Likely) benign		(Likely) pathogenic		LR towards	(95% confidence
Frequency category	n	%	n	%	pathogenicity	interval)
Single observation	59	13.32	8	8.99	0.67	(0.33–1.36)
≥ 0.0001 & < 0.01	110	24.83	0	1.12^b^	0.05	(0.01–0.32)
> 0 & < 0.0001	119	26.86	3	3.37	0.13	(0.04–0.39)
Not observed	155	34.99	78	87.64	2.50	(2.16–2.91)
*Total*	443		89			

^a^ See methods for details of control datasets and assignment of frequency category.

^b^ Percentage is calculated assuming a single variant in this category, and thus provides a conservative estimate of the LR.

Overall, these findings, based on empirical data, have utility to inform the ongoing adjustment of the ENIGMA *BRCA1/2* guidelines. Use of these frequency cut‐offs for variants designated in this study (Table S1) as Class 3 Uncertain or with posterior probability not calculated, suggests strong evidence against pathogenicity for 98 variants, moderate evidence against pathogenicity for 147 variants, and supporting evidence in favor of pathogenicity for 468 variants. It is relevant to acknowledge that some variants detected in public databases ‐ and also in clinical datasets ‐ may be somatic rather than germline in origin, arising due to clonal hematopoiesis of indeterminate potential. However, the proportion of variants arising due to this mechanism is expected to be rare for *BRCA1* and *BRCA2* (estimated as < 0.2% of all pathogenic variants in one study of >200,000 cancer gene tests (Coffee et al., 2017)), and we would also anticipate that the majority of such variants would be filtered out by generic allele fraction cut‐offs used by sequencing pipelines set up to detect true germline variants.

### 3.4 Caveats, considerations, and conclusions

This study is the largest presentation of multifactorial likelihood analysis to date. Although we mined existing public data and requested clinical data for calculations from more than 300 individuals on the ENIGMA mailing list, we recognize that the classifications assigned may alter with addition of data from other sources, and/or with the application of qualitative classification criteria. Efforts to collate information in a transparent manner that retains patient confidentiality, and within the bounds of ethical constraints, remain a challenge. As one step towards transparency, we present summary estimates of LRs for the individual components included in the analysis, and the sources of the different information types. Further discussion, and probably technical developments, will be required to determine how more detailed information, for example, segregation scores for individual families, may be presented for future large‐scale studies.

A prepublication iteration of the classification dataset was used as a reference set for the Critical Assessment of Genome Interpretation (CAGI) 5 experiment, results from which are presented in this same *Journal* issue (Cline et al., [Link]). Comparison of various different prediction methods from six different teams highlighted that prediction of mRNA splicing is an important inclusion in variant interpretation algorithms, and also indicated that variant interpretation may be improved by incorporating amino acid accessibility as a component of bioinformatic prediction of variant effect. It also showed that use of clinical information, when available, provides significant improvements to variant classification over purely bioinformatic approaches. In addition to the CAGI 5 experiment, we chose to use an updated dataset for calibration of isolated data types commonly used as components of qualitative classification approaches. Specifically, the variant classifications from multifactorial likelihood analysis were derived without use of laboratory splicing and functional data, or variant frequency in outbred reference populations. This allowed us to estimate independent LRs for or against pathogenicity for these evidence types, with several purposes: to assess the validity of ACMG/AMP code strengths proposed for these evidence types when applying them to classification of *BRCA1/2* variants; to justify specific ENIGMA *BRCA1/2* classification criteria incorporating population frequency or mRNA splicing data; to provide guidance on incorporation of BRCA1/2 protein functional assay data, to assess *BRCA1/2* predicted missense variants specifically, in quantitative or qualitative (rules‐based) classification models. We acknowledge that further analyses are necessary before such LR estimations be formally reviewed and incorporated into guidelines for *BRCA1/2* Expert Panel variant interpretations. Namely, it will be important to investigate LR estimations with additional variants that have been previously assessed using the multifactorial likelihood approach, and to derive LRs separately for *BRCA1* and *BRCA2* given differences in the penetrance for truncating variants in these two genes and the potential for differences in sensitivity and specificity of different laboratory assays to detect impact on function. Nevertheless, we note that, despite the fact that known *BRCA1/2* pathogenic variants are seen in reference population datasets, and acknowledging reservations that absence in control populations overall is not a predictor of variant pathogenicity, it appears that this feature does have value for examining the clinical relevance of rarer variants presenting for assessment at this point in time; that is, accounting for the fact that “known pathogenic” variants observed in control datasets are more likely to have already been observed in the clinical setting and already classified using other information types. This observation highlights issues around constancy of evidence strengths over time, and indicates that it will be important to reconsider such analysis periodically to re‐estimate LRs and corresponding rule strengths as the pool of variants remaining to be classified alters over time. Following this line of thought, it will be important that estimates of prior probability based on bioinformatic predictions are re‐estimated using updated datasets that reflect changed variant pools, and altered patient ascertainment in the era of multigene panel testing. Results arising from the CAGI 5 experiment (Cline et al., [Link]), and other similar studies, are likely to inform development of such bioinformatic methods.

In summary, we have used the multifactorial likelihood analysis approach to generate 248 new or considerably altered *BRCA1/2* variant classifications, information that is relevant for medical management – including determining patient eligibility for screening or PARPi treatment, and cascade testing of their relatives. We have also shown the value of this dataset for confirming existing ClinVar assertions, and for calibration of additional data types useful for variant interpretation. We have provided as supplementary information details regarding data sources and likelihood scores for all variants investigated, so providing a resource that will facilitate continued assessment of variants as additional information accrues, and further calibration of new lines of evidence relevant for variant interpretation.

## FUNDING

kConFab and kConFab Clinical Follow Up Study: National Breast Cancer Foundation (Australia), National Health and Medical Research Council (NHMRC), Queensland Cancer Fund, Cancer Councils of New South Wales, Victoria, Tasmania and South Australia, Cancer Foundation of Western Australia, and Cancer Australia.

BCAC and iCOGS: Cancer Research UK (grant numbers C1287/A16563, C1287/A10118, C1287/A10710, C12292/A11174, C1281/A12014, C5047/A8384, C5047/A15007, C5047/A10692, C8197/A16565), the European Unions Horizon 2020 Research and Innovation Programme (grant numbers 634935 and 633784 for BRIDGES and B‐CAST respectively), the European Communitys Seventh Framework Programme under grant agreement no. 223175 (HEALTHF2–2009‐223175) (COGS), the National Institutes of Health (CA128978) and Post‐Cancer GWAS initiative (1U19 CA148537, 1U19 CA148065‐01 (DRIVE), and 1U19 CA148112 ‐ the GAME‐ON initiative), the Department of Defence (W81XWH‐10‐1‐0341), and the Canadian Institutes of Health Research CIHR) for the CIHR Team in Familial Risks of Breast Cancer (grant PSR‐SIIRI‐701).

QIMR Berghofer Medical Research Institute: MT Parsons is supported by a grant from Newcastle University, UK E Tudini was supported by a grant from the National Health and Medical Research Council (NHMRC, ID1104808). AB Spurdle is supported by NMHRC Senior Research Fellowship ID 1061778.

Barretos Cancer Hospital: The research of EI Palmero was supported by Barretos Cancer Hospital, FINEP ‐ CT‐INFRA (02/2010). EI Palmero and RM Reis receive a National Council of Technological and Scientific Development (CNPq) scholarship.

Dutch/Belgian Consortium: The work of MPG Vreeswijk was financially supported by the Dutch Cancer Society KWF (UL2012–5649 and KWF/Pink Ribbon‐11704). SMoghadasi was supported by the Netherlands Organization for Scientific Research (NWO), research program Mosaic (Grant 017.008.022) and Van de Kampfonds from Leiden University Medical Centre (Grant 30.925).

French UNICANCER Genetic Group (http://www.unicancer.fr/recherche/les‐groupes‐recherche/groupe‐genetique‐et‐cancer‐ggc): S. M. Caputo is supported by the French National Institute of Cancer for the curation of the *BRCA1/BRCA2/PALB2* variant database. Astra Zeneca contributes financially to the COVAR study.

Fundación Pública Galega Medicina Xenómica: A. Vega is supported by the Spanish Health Research Foundation, Instituto de Salud Carlos III (ISCIII), partially supported by FEDER funds through Research Activity Intensification Program (contract grant numbers: INT15/00070, INT16/00154, and INT17/00133), and through Centro de Investigación Biomédica en Red de Enferemdades Raras CIBERER (ACCI 2016: ER17P1AC7112/2018); the Autonomous Government of Galicia (Consolidation and structuring program: IN607B), and by the Fundación Mutua Madrileña (call 2018).

German Consortium for Hereditary Breast and Ovarian Cancer: GC‐HBOC is funded by the German Cancer Aid (#110837, #70111850; coordinator: RK. Schmutzler). L Wiesmullerreceived PhD fellowships from the International Graduate School in Molecular Medicine Ulm University.

Hospital Clinico San Carlos: M de la Hoya is supported by the European Union's Horizon 2020 Research and Innovation Program under grant agreement No 634935, and Spanish Instituto de Salud Carlos III (ISCIII) grant PI15/00059, an initiative of the Spanish Ministry of Economy and Innovation partially supported by European Regional Development FEDER Funds.

Helsinki University Hospital: The work of H Nevanlinna was supported by the Helsinki University Hospital Research Fund.

Huntsman Cancer Institute, University of Utah: Sean Tavtigian acknowledges support from the National Institutes of Health (CA128978 and CA121245).

ICCon Partnership: ICCon acknowledges input from Miriam Fine, Melissa Monnik, Rachel Austin, Letitia Thrupp, Chris Michael‐Lovatt and Gillian Mitchell towards ICCon co‐ordination, data collection and curation. The ICCon Partnership is funded by the Cancer Council New South Wales Strategic Research Partnership (STREP) scheme.

Institut Català d'Oncologia: The work of C Lazaro is supported by the Carlos III National Health. The Institut Català d'Oncologia is funded by FEDER funds – a way to build Europe – (PI16/00563 and CIBERONC), the Government of Catalonia (Pla estratègic de recerca i innovació en salut [PERIS_MedPerCan and URDCat projects], 2017SGR1282 and 2017SGR496), and the Scientific Foundation Asociación Española Contra el Cáncer.

Mayo Clinic: The research of F Couch was supported by NIH Specialized Program of Research Excellence (SPORE) in breast cancer (P50 CA1162091), NIH grants CA116167 and CA192393, and the Breast Cancer Research Foundation.

Moffit Cancer Centre: A Monteiro acknowledges support from the NIH (CA116167).

New Zealand Familial Breast Cancer Study: L Walker was supported by the Rutherford Discovery Fellowship (Royal Society of New Zealand). V Lattimore was supported by the Breast Cancer Foundation of New Zealand Belinda Scott Clinical Fellowship.

NICEST (Network of Italian Collaborators to ENIGMA Studies and Trials): NICEST is partially supported by funds from the Italian Association of Cancer Research (AIRC, Individual grant 15547, to P. Radice).

Ohio State University: The research of A Toland was supported by the Ohio State University Comprehensive Cancer Center.

Pisa University Hospital‐SOD Genetica Molecolare: Maria A Caligo is supported by a grant from Fondazione Pisa (Grant “Clinical characterization of BRCA 1/2 Missense variants for evaluation of Breast cancer Risk”, 2018–2020).

University of Manchester: DGR Evans and ER Woodward are funded by the NIHR Manchester Biomedical Research Centre (IS‐BRC‐1215–20007), and ER Woodward is supported by the Cancer Research CRUK Catalyst Award, CanGene‐CanVar (C61296/A27223).

Vall d'Hebron Institute of Oncology (VHIO): This study was supported by Spanish Instituto de Salud Carlos III (ISCIII) funding, an initiative of the Spanish Ministry of Economy and Innovation partially supported by European Regional Development FEDER Funds (FIS PI15/00355 to O. Diez, FIS PI13/01711 and FIS PI16/01218 to S. Gutiérrez‐Enríquez). S. Gutiérrez‐Enríquez is supported by the Miguel Servet Program from ISCIII (CPII16/00034).

## CONFLICT OF INTERESTS

The following authors declare conflicts as stated below.

Sandrine M. Caputo: Astra Zeneca contributes financially to the COVAR study.

Laura Cortesi: Astra Zeneca (honoraria), Pfizer (honoraria and advisory role), Amgen (advisory role), and Novartis (advisory role).

Fergus Couch: Steering committee and diagnostics committee for the Astra Zeneca LUCY study.

Anne‐Marie Gerdes: February 2016, advisory Board meeting about BRCA1/2 testing in ovarian cancer, sponsored by Astra Zenica.

Paolo Radice: Scientific coordinator of a course on the classification of BRCA gene allele variants sponsored by Astra Zeneca (Milan, June 2018).

Angela Toss: Lilly (advisory role), Roche (advisory role).

Lisa Wiesmuller is an inventor of a patent on a test system for determining genotoxicities, which is owned by LW.

Ana Vega: October 2015, advisory Board meeting about BRCA1/2 testing in ovarian cancer, sponsored by Astra Zeneca.

All other authors declare that they have no conflict of interests.

## Supporting information

Supplementary information
